# Mammary and Extramammary Paget's Disease Presented Different Expression Pattern of Steroid Hormone Receptors

**DOI:** 10.1155/2017/3768247

**Published:** 2017-09-10

**Authors:** Songxia Zhou, Weixiang Zhong, Ruiqin Mai, Guohong Zhang

**Affiliations:** ^1^Department of Pathology, Shantou University Medical College, Shantou, Guangdong, China; ^2^Department of Laboratory Medicine, The First Affiliated Hospital of Shantou University Medical College, Shantou, Guangdong, China; ^3^Department of Pathology, The First Affiliated Hospital, College of Medicine, Zhejiang University, Hangzhou, Zhejiang, China

## Abstract

**Background and Objectives:**

Paget's disease (PD) is a rare intraepithelial adenocarcinoma, which is composed of mammary (MPD) and extramammary Paget's disease (EMPD). Currently, the published literature contains scant data on expression pattern of steroid hormone receptors in MPD and EMPD.

**Methods:**

Expression of estrogen receptor (ER) and androgen receptor (AR) was evaluated in 88 MPD and 72 EMPD by using immunohistochemical staining and H-score method.

**Results:**

Positive expression of AR was significantly higher in EMPD (61.11%, 44/72) than in MPD (32.95%, 29/88) (*P* < 0.001), while ER expression was positive 19.44% (14/72) in EMPD and only 9.09% (8/88) in MPD (*P* = 0.059). ER-AR expression pattern was significantly different between MPD (3.41%, 3/88) and EMPD (16.67%, 12/72) (*P* < 0.001). No difference of AR (*P* = 0.301) or ER (*P* = 0.239) expression was identified between invasive (48.57%, 51/105 of AR, and 11.43%, 12/105 of ER) and noninvasive PD. In MPD, no difference of AR expression between MPD alone (7/18, 38.89%) and MPD with underling ductal carcinoma of breast (22/70, 31.43%) was identified (*P* = 0.548). In EMPD, expression of AR was 63.33% (38/60) in penoscrotal EMPD.

**Conclusion:**

Our current results indicate that MPD and EMPD presented different expression pattern of AR and ER and would help to further identify the molecular subtype of MPD and EMPD for adjuvant hormonal therapy, especially for patients with penoscrotal EMPD.

## 1. Introduction

Paget's disease (PD) is a rare cutaneous intraepithelial malignancy characterized by large adenocarcinoma cells containing abundant mucin and has two subtypes according to the affected anatomic location: mammary Paget's diseases (MPD) and extramammary Paget's diseases (EMPD). Epidemiologically, MPD is accounting for 1–4.3% of all primary breast carcinoma [1], among that 93–100% of MPD associated with underlying ductal carcinoma of breast [2], while EMPD predominantly affects apocrine gland-bearing areas, such as vulva in female (81.3%) and scrotum in male (43.2%), respectively [3]. The incidence rates of EMPD increasing with an annual percent change of +3.2% since 1978, such as scrotum EMPD, were accounting 21% of primary scrotal carcinoma [4]. Interestingly, those EMPD affected organs including vulva in female and scrotum in male, which are the hormonal-targeted tissues.

Although the incidence of vulvar EMPD is rare, several reports described heterochronous development of EMPD in the vulva and MPD [5], even synchronous development of EMPD of the vulva and MPD lesions [6–9]. Furthermore, performance status of patient with ER-positive EMPD was maintained after treating with ER-inhibitor tamoxifen. This successful evidence suggested that hormonal therapy may be an alternative for selected cases of advanced EMPD [10]. Therefore, hormonal receptors might be the link between MPD and EMPD and may be a way of understanding their common pathogenesis.

Previously, AR-positive rates of 88% in MPD and 78% in EMPD were described by Liegl et al. [11]; however, positive rate of AR expression only 54–57% in EMPD had been recorded in literature as well [12, 13]. Particularly, there are few documented studies of ER and AR expression of MPD and EMPD in China. The positive rate of AR and ER in MPD and EMPD still remains scant and needs to be further validated in a big sample set. Here, we present ER and AR expression in a larger series of MPD and EMPD by immunohistochemistry.

## 2. Materials and Methods

### 2.1. Samples and Histologic Evaluation

Multicentre collaboration was established to recruit archival paraffin embedded biopsies for sample blank. All MPD and EMPD samples were routine stained by HE and were evaluated separately by two independent pathologists (Songxia Zhou and Guohong Zhang). Invasive EMPD was histologically defined as Paget cells infiltrated in the dermis. The study procedure was approved by the Institutional Ethics Board of Shantou University Medical College.

### 2.2. Immunohistochemistry and Assessment

Paraffin sections (4-5 *μ*m) were subjected to immunohistochemistry for ER and AR. The immunohistochemistry staining was performed as described previously [14]. Briefly, primary rabbit monoclonal anti-ER antibody (Maixin Biotech Co., Fuzhou, China) and mouse monoclonal anti-AR antibody (ZSGB-BIO Co, Beijing, China) were used. The negative control was set by PBS instead of primary antibody, with all other conditions kept the same. Immunohistochemical staining was scored based on the percentage and intensity of the stained cells. The staining percentage was scored from 0% to 100%, while the staining intensity was scored from 0 through 3 (0 = negative, 1 = weak, 2 = moderate, and 3 = strong). Intensity score was multiplied by the percentage of cells displaying that intensity using the following formula: (0 × % cells 0) + (1 × % cells 1) + (2 × % cells 2) + (3 × % cells 3), to yield an H-score ranging from 0 to 300. Based on this semiquantitative scoring system of H-score, we used ROC curve to define the optimal cutoff point for the negative and positive scores for ER and AR expression.

### 2.3. Statistical Analysis

Data were analyzed using SPSS software for Windows, version 16.0 (SPSS Inc., Chicago, IL). Two-sample *t*-tests were used to compare continuous variables, while Chi-square tests or Fisher's exact tests were used to evaluate the difference between categorical variables. *P* < 0.05 was considered to reflect statistical significance.

## 3. Results

Finally, 88 MPD and 72 EMPD could be used for simultaneous analysis of ER and AR.  Table 1 summarized characteristics of the study cohort. In this cohort, mean age was significantly younger in MPD (ranging from 23 to 80 years) than that in EMPD (ranging from 46 to 90 years), and only 3 of 72 cases were associated with underlying malignancy in EMPD and lower than that in MPD (79.55%, 70/88).

**Figure 1:**
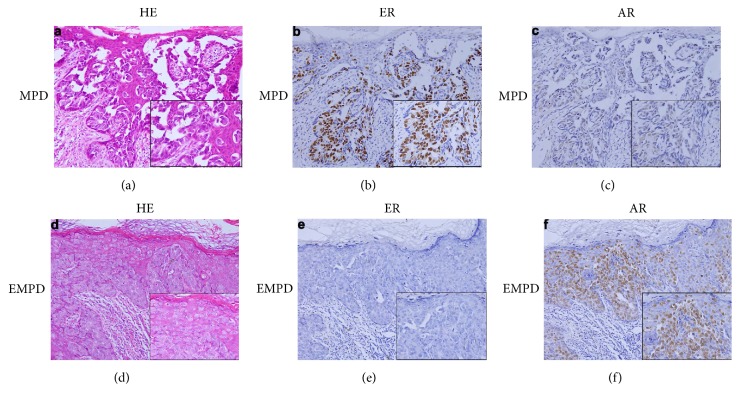
Representative images of histological and immunohistological features of MPD and EMPD. (a) HE staining of MPD, (b) nuclear positive staining of ER in MPD, (c) nuclear negative staining of AR in MPD, (d) HE staining of EMPD, (e) negative staining of ER in EMPD, and (f) nuclear positive staining of AR in EMPD.

Representative results of immunohistochemical staining for ER and AR are shown in Figure 1. Totally, ER- and AR-positive were seen in 13.75% (22/160) and 45.63% (73/160) PD, respectively (Table 2). The morphological feature of signet-ring cell carcinoma and adenocarcinoma of apocrine had been identified in our sample cohort, and no significant difference of expression of ER (*P* = 0.496) or AR (*P* = 0.514) between the 19 signet-ring cell carcinomas and 141 adenocarcinomas of apocrine, respectively. No difference of AR (*P* = 0.301) or ER (*P* = 0.239) expression was observed between invasive (48.57%, 51/105 of AR, and 11.43%, 12/105 of ER) and noninvasive PD (40%, 22/55 of AR, and 18.18%, 10/55 of ER).

Then, according to anatomic location, the expression rates of AR were significantly higher in EMPD (61.11%, 44/72) than in MPD (32.95%, 29/88) (*P* < 0.001, Table 2). However, ER expression was positive 19.44% (14/72) in EMPD and only 9.09% (8/88) in MPD (*P* = 0.059), respectively. After combination of ER and AR, coexpression of ER and AR was found in 12 out of 72 (16.67%) EMPD cases and only in 3 out of 88 (3.41%) MPD cases, the ER-AR expression pattern was significantly different between MPD and EMPD (*P* < 0.001, Table 3). No association of AR expression and invasion status in EMPD was observed with 60.87% in noninvasive and 61.22% in invasive cases (*P* = 0.977).

For specific subgroup, among the 88 MPD, no significant differences (*P* = 0.548) of AR expression were observed between MPD alone (7/18, 38.89%) and MPD with underling ductal carcinoma (22/70, 31.43%). Because majority of EMPD were localized in the penoscrotal area, we found the AR and ER expression were positive, 63.33% and 20%, and yield a coexpression of ER and AR in 11 out of 60 penoscrotal EMPD cases.

## 4. Discussion

James Paget first described MPD in 1874, and EMPD was first described by Radcliffe Crocker in 1889. For the rarity of MPD and EMPD in clinical practice, in the past 120 years, few researchers have paid enough attention to the molecular profile differently involved in MPD and EMPD, although MPD and EMPD have similar clinical features and identical histological morphology.

Firstly, our results described the different expression AR in EMPD (61.11%) than in MPD (32.95%), which suggested that AR is putative link MPD and EMPD and may be a common biomarker for partial MPD and EMPD. On the other side, for the accurate AR expression rate, positive rate of 84% (42/50) [15], 78% (18/23) [11], 57% (33/58) [13], and 54% (15/28) [12] in EMPD had been described in literature. However, the sample size was limited. In this study, AR expression was positively presented in 44 out of 72 cases, and our data confirmed that the positive rate of AR in EMPD was close to 55 ± 5% and far away from 70% and 80%. AR has biological and therapeutic utilization in prostate carcinoma, but its use in EMPD treatment is rarely reported because of few evidence of AR expression in EMPD. From our evidences, the AR expression could be used for selecting subgroup of EMPD, and AR antagonists might provide benefit to EMPD patients with AR-positive expression. Particularly for EMPD in penoscrotal area, because the common initial sites of EMPD are the scrotum of male in China [16], our data indicated that the 63.33% of EMPD with AR expression in penoscrotal area should be selected for further adjuvant hormonal therapy. Similarly to previous frequencies of AR expression in noninvasive (24/42, 57%) and invasive (9/16, 56%) EMPD described by Kasashima et al. [13], in our study, no association of AR expression and invasion status in EMPD was observed with 60.87% in noninvasive and 61.22% in invasive cases (*P* = 0.977).

No significant difference of ER expression in EMPD (19.44%) and MPD (9.09%) was observed in this study. Compared with data in literature, the positive rate of 19.44% in EMPD was higher than previous rate of 4% (1/23) by Liegl et al. [11] and none by De Leon et al. [12]. However, the 9.09% of MPD were ER-positive in our study that is similar to the 10% ER-positive in MPD found by Liegl et al. [11]. Combined together, current data confirm that the ER was rarely detected in EMPD and MPD as well.

The histogenesis of MPD is controversial; several hypotheses have been proposed. Epidermotropic theory is a main hypothesis, which stated that Paget cells would be cells from the generally present underlying intraductal cancer that migrated through the basement membrane to the nipple [2]. Our results suggested that no significant differences of AR expression were observed between MPD alone (38.89%) and MPD with underling ductal carcinoma (31.43%). To the best of our acknowledge, this is the first time to compare directly between MPD with and MPD without breast carcinoma, suggest that MPD alone and MPD underling with breast carcinoma have common histogenesis, and provide the clue to support the fact that MPD is not a simple consequence of breast carcinoma invasion.

Finally, up to date, only 4 publications of case report for primary PD with signet-ring cell carcinoma are recorded in literature [17–20]. Our data also demonstrate no difference of AR expression between 17 signet-ring cell carcinomas and adenocarcinomas of apocrine. Signet-ring cell carcinomas are neoplastic cells with abundant cytoplasm eccentrically located with nuclei which are seen mostly in stomach and colorectum. Our data suggest that signet-ring cell carcinomas shared the common molecular basis with adenocarcinomas of apocrine.

## 5. Conclusion

Together, our results described expression pattern of steroid hormone receptors and provide convincing evidence for a potential histogenetic link between MPD and EMPD and AR inhibitor as hormonal therapy for EMPD.

## Figures and Tables

**Table 1:** Clinic pathological characteristics of patients and tumors.

	MPD (*N* = 88)	EMPD (*N* = 72)
	Number (%)	Number (%)
Primary location		
Nipple	88 (100)	0 (0)
Perianal	0 (0)	6 (8.33)
Penoscrotal	0 (0)	60 (83.33)
Inguinal	0 (0)	2 (2.78)
Axillary	0 (0)	1 (1.40)
Perineal	0 (0)	3 (4.16)
Gender		
Male	0 (0)	68 (94.44)
Female	88 (100)	4 (5.56)
Age	54.93 ± 10.57	65.72 ± 9.78
Invasive status		
Carcinoma in situ	32 (36.36)	23 (31.94)
Invasive carcinoma	56 (63.64)	49 (68.06)
Histopathological type		
Signet ring cell carcinoma	6 (6.82)	13 (18.06)
Adenocarcinoma of apocrine	82 (93.18)	59 (81.94)
Underling carcinoma		
Present	70 (79.55)	3 (4.17)
Absent	18 (20.45)	69 (95.83)

**Table 2:** Comparison of expression levels of biological markers between MPD and EMPD.

	ER		AR	
	Positive	Negative	Positive	Negative
	Number (%)	Number (%)	Number (%)	Number (%)
MPD	8 (9.09)	80 (90.91)	29 (32.95)	59 (67.05)
MPD without UBDC	1 (5.56)	17 (94.44)	7 (38.89)	11 (61.11)
MPD with UBDC	7 (10)	63 (90)	22 (31.43)	48 (68.57)
EMPD	14 (19.44)	58 (80.56)	44 (61.11)	28 (38.89)
Penoscrotal	12 (20)	48 (80)	38 (63.33)	22 (36.67)
Perianal	1 (16.67)	5 (83.33)	1 (16.67)	5 (83.33)
Perineal	0 (0)	3 (100)	3 (100)	0 (0)
Inguinal	0 (0)	2 (100)	1 (50)	1 (50)
Axillary	1 (100)	0 (0)	1 (100)	0 (0)

^*∗*^Significant difference compared with normal (normal skin tissue from necropsy specimen); UBDC: underling ductal carcinoma of breast.

**Table 3:** Correlations between expression levels of biological markers between MPD and EMPD.

	MPD	EMPD
	Number (%)	Number (%)
ER/AR		
+/+	3 (3.41)	12 (16.67)
+/−	5 (5.69)	2 (2.78)
−/+	26 (29.54)	32 (44.44)
−/−	54 (61.36)	26 (36.11)
